# Optimal Tuning Perspective of Range-Separated Double Hybrid Functionals

**DOI:** 10.1021/acs.jctc.2c00082

**Published:** 2022-04-02

**Authors:** Georgia Prokopiou, Michal Hartstein, Niranjan Govind, Leeor Kronik

**Affiliations:** † Department of Molecular Chemistry and Materials Science, Weizmann Institute of Science, Rehovoth 76100, Israel; ‡ Physical and Computational Sciences Directorate, Pacific Northwest National Laboratory, Richland, Washington 99352, United States

## Abstract

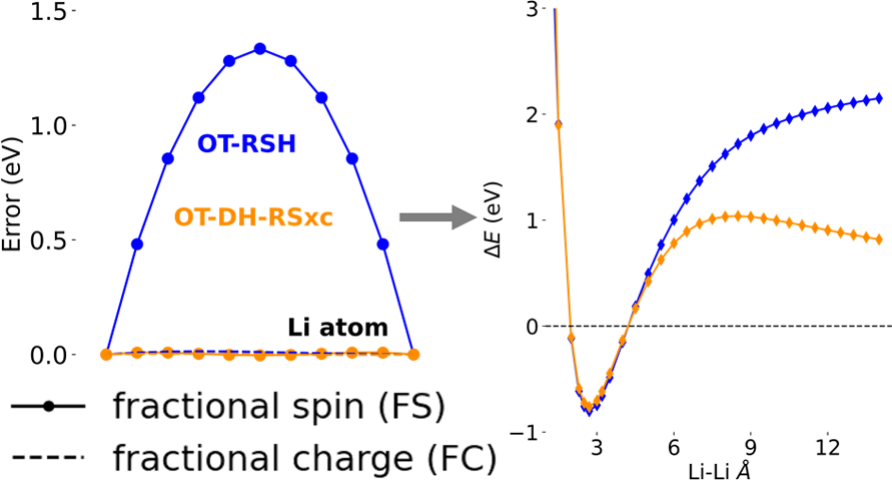

We study the optimal tuning of the free parameters in range-separated double hybrid functionals, based on enforcing the exact conditions of piecewise linearity and spin constancy. We find that introducing the range separation in both the exchange and the correlation terms allows for the minimization of both fractional charge and fractional spin errors for singlet atoms. The optimal set of parameters is system specific, underlining the importance of the tuning procedure. We test the performance of the resulting optimally tuned functionals for the dissociation curves of diatomic molecules. We find that they recover the correct dissociation curve for the one-electron system, H_2_^+^, and improve the dissociation curves of many-electron molecules such as H_2_ and Li_2_, but they also yield a nonphysical maximum and only converge to the correct dissociation limit at very large distances.

## Introduction

Density functional theory (DFT)^1,2^ has long been the workhorse for first-principles calculations in the fields of physics, chemistry, and materials science.^3−9^ DFT is an exact theory in principle, but as it requires an exchange–correlation (xc) energy expression that is generally unknown, it is almost always approximate in practice.

Among the many forms of approximate xc functionals, orbital-dependent functionals have long been known to offer additional flexibility in functional construction that can be translated into improved accuracy at a reasonable computational cost.^10^ In particular, global hybrid functionals,^11−13^ which incorporate a fraction of nonlocal exact or Hartree–Fock (HF) exchange (and are formally part of the fourth rung of the “Jacob’s ladder”^14^ functional classification system), have found widespread use. A more sophisticated class of hybrid functionals are the range-separated hybrid (RSH) functionals.^15,16^ In this approach, the electron–electron interaction is partitioned into short-range (SR) and long-range (LR) contributions, allowing for different exchange treatments in the two ranges. For molecules, often full HF exchange is used for the LR part, which restores the correct asymptotic potential, and a mixture of semilocal and HF exchange is used for the SR part, which retains the advantages of the global hybrid functional in balancing SR exchange with correlation. This allows RSH functionals to be asymptotically correct, to mitigate self-interaction errors, and to mitigate or sometimes even eliminate localization/delocalization errors,^17^ resulting often in excellent performance.

Clearly, not all issues can be resolved by improving the treatment of exchange. Many remaining shortcomings in the accuracy of xc functionals^18−22^ call for an orbital-dependent expression not just for exchange but also for correlation. One popular approach for implementing this idea, in practice, is the use of double hybrid (DH) functionals,^23,24^ where both a fraction of exact exchange and a fraction of second order Møller–Plesset (MP2)^25^ correlation are admixed. Such DH functionals are part of rung 5 of “Jacob’s ladder”, as the MP2 correlation expression requires unoccupied or virtual orbitals. DH functionals have been shown to yield improved results for many challenging cases, e.g., van der Waals interactions,^26,27^ spin-state energetics,^28,29^ and generally an improved treatment of thermochemistry.^30−32^

The RSH idea can be combined with the DH concept in two different ways. The simpler way is to use a range-dependent admixture of HF exchange together with a global admixture of MP2 correlation,^33−40^ an idea that has already resulted in more accuracy. A more general approach is to use an RSH scheme where *both* exchange and MP2 correlation are range-separated.^41^ Specific parametrizations of this general scheme were already shown to improve the treatment of van der Waals interactions,^42−44^ fractional-charge scenarios,^45^ and excited states.^46,47^ We note that while the two approaches are generally not the same, both are often referred to in the literature as a “range-separated double hybrid”. To avoid confusion, we refer to the former as “DH-RSx” and to the latter as “DH-RSxc” in the rest of this paper.

A crucial step for the accuracy of a single RSH functional (i.e., where range separation is used only for the exchange term) is the choice of the range-separation parameter. In the optimally tuned RSH (OT-RSH) functional,^48−57^ the range-separation parameter is chosen from first principles, based on satisfaction of the ionization potential (IP) theorem for each system separately. OT-RSH has been shown to be highly successful in eliminating fractional charge errors^58^ and mitigating large self-interaction errors.^59,60^ It is therefore interesting to generalize the optimal tuning idea to DH functionals and to examine whether this is advantageous. This introduces more free parameters. Here we examine the importance of tuning these parameters based on two exact conditions:^61−64^ the piecewise linearity condition^65,66^ and the spin constancy condition.^67^ Using a generalization of MP2,^68^ for the illustrative case of singlet atoms, we find that the DH-RSxc scheme offers an advantage over the DH-RSx scheme as it allows the minimization of both errors with the same set of parameters. For dissociation curves of simple diatomic molecules, we find significant improvement compared with single hybrid and some DH functionals. The correct dissociation limit is also obtained at large distances, but a nonphysical maximum is still found for intermediate distances.

## Theory

In RSH functionals, the Coulomb repulsion is split, often using the error function in the form^69^
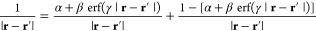
1If HF and semilocal exchange are used to evaluate the first and second terms in eq 1, respectively, then the exchange (x) energy is split into long-range (LR) and short-range (SR) terms,^16^ in the form^69^
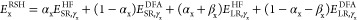
2where we use the superscripts HF and DFA (density functional approximation) to denote the type of exchange treatment and the subscript “x” for the parameters α, β, and γ to emphasize that the exchange is range-separated. For molecules, the OT-RSH scheme chooses α_x_ + β_x_ = 1 in order to recover the correct asymptotic decay of the xc potential.^70,71^ The range-separation parameter γ_x_ is tuned so as to satisfy the IP theorem,^51,72^ which states that the eigenvalue of the highest occupied molecular orbital (HOMO) of the system with *N* electrons is equal to the IP, which is the total energy difference of the *N* and *N* – 1 electron systems:^65,73,74^
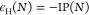
3

In this way, α_x_ is the only free parameter in eq 2 and it is usually set to α_x_ = 0.2, which is similar to the exchange fraction typically used in global hybrid functionals.^12,13^

In single RSH functionals, the exchange expression of eq 2 is augmented with standard semilocal correlation. In DH-RSx functionals, one admixes a global fraction, α_c_, of MP2 correlation to the functional, yielding
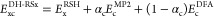
4In eq 4, *E*_c_^MP2^ is the MP2 correlation energy, given by
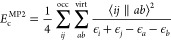
5where ϵ_*i*_ is the eigenvalue of the *i*th orbital and ⟨*ij*||*ab*⟩ = ⟨*ij*|*ab*⟩ – ⟨*ij*|*ba*⟩, with
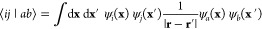
The spin–orbital is defined as ψ_*i*_(**x**) = ϕ_*i*_(**r**) ξ_*i*_(*s*), where ξ_*i*_(*s*) = α(*s*) or β(*s*) for spin up or spin down, respectively, and ϕ_*i*_(**r**) is a real spatial orbital.

Equation 4 contains four free parameters: α_x_, β_x_, γ_x_, and α_c_. ωB2PLYP,^36^ ωB2GPPLYP,^36^ RSX-QIDH,^34^ and RSX-0DH^35^ are four examples of DH-RSx functionals that are all special cases of eq 4, obtained using different choices for the semilocal DFA and the above parameters.

In general, the MP2 term in eq 5 diverges if the energy gap between occupied and virtual states vanishes. For such cases, we follow the work of Cohen et al.,^75^ who instead used the degeneracy-corrected perturbation theory (DCPT2) expression:^68,76^
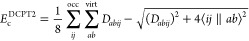
6where *D*_*abij*_ = ϵ_*a*_ + ϵ_*b*_ – ϵ_*i*_ – ϵ_*j*_. For nondegenerate cases, DCPT2 yields almost the same results as MP2 but overcomes the divergence (see refs (68) and (75) and Figure I.1 in the Supporting Information).

In a DH-RSxc functional, a more general scheme with range-separated MP2 (RS-MP2) correlation,^41^ which again can be replaced by DCPT2, is used, leading to
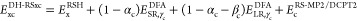
7where
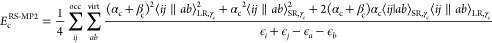
8
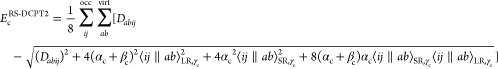
9

In eqs 8 and 9
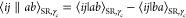
with
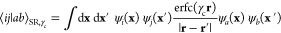
and
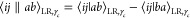
with
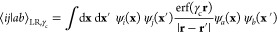
(See ref (41) and section I in the Supporting Information for the derivation of RS-MP2.) In the most general case, eq 7 contains six free parameters: α_x_, β_x_, γ_x_, α_c_, β_c_, and γ_c_.

One way to reduce the number of free parameters in a nonempirical manner is to apply constraints based on known exact conditions that an xc functional should obey. In this work, we choose α_x_ + β_x_ = 1 and α_c_ + β_c_ = 1 throughout, in order to obtain the correct asymptotic behavior of the exchange and correlation potentials.^73^ Beyond asymptotic behavior, a general rule that the exact xc functional must obey is the flat-plane condition.^63,64,77,78^ This condition specifies that the energy of a general system, if plotted as a function of both fractional charge and fractional spin, will produce two flat planes intersecting in a seam defined by a line of constant and integer electron number.^77,79^ Two simple special cases of this general condition are the piecewise-linearity condition, which specifies that the total energy is piecewise-linear for a system with fractional charge and constant spin,^65,66^ and the spin-constancy condition,^67^ which specifies that the total energy is constant for a system with constant charge but varying spin. We will refer to deviation from these conditions as fractional charge (FC) and fractional spin (FS) errors, respectively, defined as
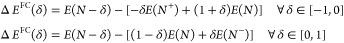
10and
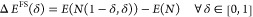
11

Here, we extend optimal tuning to a DH functional by seeking a set of parameters that minimizes FC errors as in conventional optimal tuning,^58^ but also simultaneously minimizes FS errors. To that end, we need to evaluate DH energies for FC and/or FS scenarios, which requires a generalization of eqs 2, 5, and 6 to fractional occupations,^75,80^ in the form
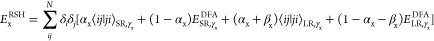
12
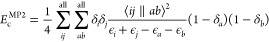
13
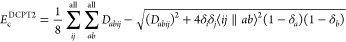
14where δ_*i*_ is the occupation number of the *i*th orbital, with a similar extension for the range-separated expressions of eqs 7–9. We note that Hirata et al.^81,82^ proposed a renormalized expression for MP2 (renorm-MP2) with fractional occupations, which restores the correct zero-temperature limit for metallic systems. Margraf and Bartlett showed^83^ that the overall shape of the FC curves generated with the conventional and the renormalized MP2 expressions are qualitatively similar but somewhat different quantitatively (see section II in the Supporting Information for more details). Owing to the qualitative similarity, this has not being pursued further here.

## Computational Details

All calculations presented in this work were performed using a locally modified version of NWchem v.6.8.1.^84^ A spin-unrestricted formalism and the cc-pvtz basis set^85,86^ were used throughout.

Equations 5, 6, 8, and 9, allowing for fractional occupations, were implemented in the semidirect algorithm^87^ to compute the MP2 or DCPT2 energy. In our current implementation, MP2 and DCPT2 energies are calculated with the converged single hybrid RSH orbitals and eigenvalues. Therefore, only the total energies are affected by the MP2 and the DCPT2 terms. We note that it is known^88,89^ that, when the MP2 expression is constructed with DFT orbitals instead of HF ones, a nonzero single-excitation term arises. We do not take this term into account in the current implementation, but it can be important for weak interactions.^90^ For the DFA, all calculations were based on the “parent” semilocal functional of Perdew, Burke, and Ernzerhof (PBE).^91^ For all RSH calculations, we used the range-separated PBE exchange functional.^92−95^ We used the PBE correlation functional for the DH-RSx and the range-separated PBE correlation functional^92−95^ for the DH-RSxc functional (see section III in the Supporting Information for details of the implementation of the range-separated PBE xc functional).

## Results and Discussion

**Figure 1:**
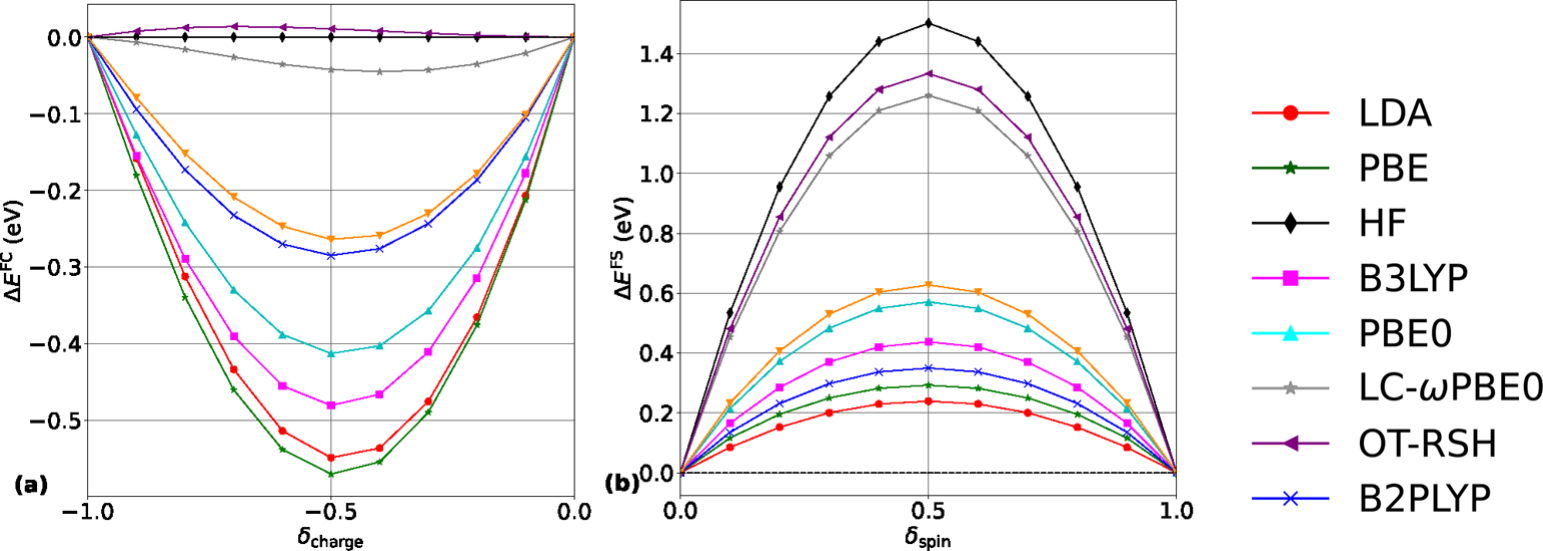
(a) FC and (b) FS errors, as defined by eqs 10 and 11, respectively, for the Li atom, calculated using LDA^96^ (−●−), PBE^91^ (green −★−), HF (−⧫−), B3LYP^11,12^ (−■−), PBE0^13^ (−▲−), LC-ωPBE0^97^ (gray −★−), OT-RSH based on LC-ωPBE0 (−◀−), B2PLYP^98^ (−×−), and PBE0-DH^99^ (−▼−).

We start our analysis by examining FC and FS errors obtained from several local and semilocal (LDA,^96^ PBE^91^), global hybrid (B3LYP,^11,12^ PBE0,^13^ and for comparison “pure” HF), single RSH (LC-ωPBE0^97^ using both the original and the optimally tuned range-separation parameter), and global double hybrid (B2PLYP^98^ (α_x_ = 0.53, α_c_ = 0.27) and PBE0-DH^99^ (α_x_ = 0.50, α_c_ = 0.125)), both used with DCPT2 on account of the fractional spin occupations) functionals, for the illustrative case of the Li atom. The results are shown in Figure 1. Figure 1a shows that LDA and PBE functionals exhibit the largest FC errors (i.e., largest deviation from piecewise linearity). These errors are reduced by global hybrid functionals, further reduced by global double hybrids, and greatly reduced by functionals with an exact asymptotic exchange. In particular, owing to the close relation between the IP theorem and piecewise linearity,^58,71^ optimal tuning reduces and almost eliminates the FC error of the parent functional. These trends are consistent with those of previous analyses.^17,58,61,62,71^

Figure 1b shows the FS error curves obtained from the same set of functionals. Interestingly, and again in agreement with past work,^61,62,100^ the FS error follows a trend essentially opposite from that of the FC curves; namely, the error is smallest for semilocal functionals and increases gradually until it is worse for HF and single RSH functionals. This opposite trend is attributed to the fact that the fractional spin system exhibits a large static correlation.^61^ This correlation^101^ is partly emulated by semilocal exchange.^102−107^ At higher rungs, the semilocal exchange content in the functionals is even lower compared to the LDA or PBE functionals, resulting in an increased FS error.

**Figure 2:**
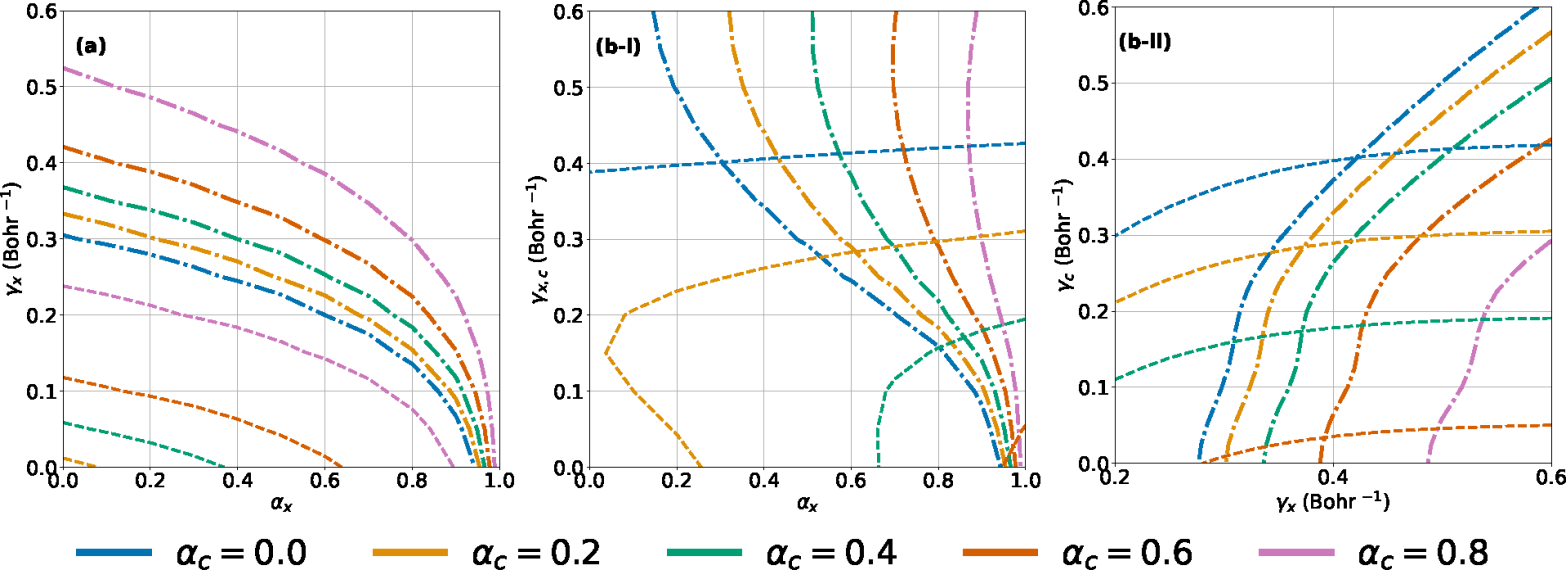
Minimum contour lines for FC (dashed–dotted lines) and FS (dashed lines) “middle point” errors equal to 10^–4^ eV for the Li atom. (a) DH-RSx (eq 4). (b) DH-RSxc (eq 7) with (i) γ_x_ = γ_c_ and (ii) α_x_ = 0.2.

One conclusion drawn from Figure 1 is that the two global DH functionals, B2PLYP and PBE0-DH, reduce the FC error compared to the (worst performing) semilocal functionals and also reduce the FS errors compared to the (worst performing) RSH and HF functionals. Importantly, it is not possible to minimize *both* errors with the same global DH functional (see section IV in the Supporting Information for more details). Obviously, a functional with higher flexibility is needed. This suggests that perhaps further improvements can be made by admixing a fraction of MP2/DCPT2 correlation based on an underlying OT-RSH functional, rather than a global hybrid functional, while optimally tuning the relevant parameters so as to minimize both FC and FS errors. Realizing this approach is more complicated than tuning of a single RSH owing to the larger number of mixing parameters in the functional. To test this idea, we first vary the parameters of a DH-RSx functional, based on range-separated PBE exchange (see eq 4) until both FC and FS errors are minimized. As clearly observed for Figure 1, the FC and FS curves usually reach their maximum for δ = ±0.5. We therefore probe the error of this middle point in the curves of Figure 1 as we vary the three parameters (α_x_, γ_x_, α_c_) of eq 4. Figure 2a provides the set of parameters that minimize the FC (dashed–dotted lines) and the FS error (dashed lines) (see section V in the Supporting Information for the individual contour plots). Evidently, it is possible to find a set of parameters for a DH-RSx that satisfy the FC or FS errors. However, Figure 2a shows that it is not possible to satisfy *both* conditions, namely minimal FC and FS errors, with the same set of parameters.

The failure of optimal tuning of the DH-RSx functional suggests that we need even more flexibility in the functional itself. An obvious way to achieve this is to extend the analysis to the DH-RSxc scheme. In fact, Kalai and Toulouse^41^ showed that adding a fraction of MP2 only in the LR (equivalent to setting α_c_ = 0.0 and α_c_ + β_c_ = 1 in eq 7) already reduces the FC error. Here, we vary the value of α_c_ and, by using DCPT2 instead of MP2, we can probe the FS error as well. We emphasize that because we use the α_c_ + β_c_ = 1 constraint, here when α_c_ = 0.0 the correlation is described solely by DFT in the SR and by MP2/DCPT2 in the LR (see eq 7). This is in contrast to the previously discussed DH-RSx case, for which α_c_ = 0.0 turns off the MP2/DCPT2 correlation altogether.

In our DH-RSxc scheme, there are four free parameters: α_x_, γ_x_, α_c_, and γ_c_. Therefore, we conduct the analysis in two ways, each constraining one parameter. In the first way, we set the range-separation parameters of the exchange and correlation parameters to be the same, i.e., γ_x_ = γ_c_. The contour lines for minimum “middle-curve” FC and FS errors obtained for this scenario are then shown in Figure 2b-i (see section VI.A in the Supporting Information for the individual contour plots). Clearly, range separating both the exchange and the correlation allows for the minimization of both FC and FS errors, which occurs at the intersection points of the respective curves in Figure 2b-i. We also conclude that α_x_ needs to be larger than α_x_ = 0.2, which is a typical value for a single hybrid, for the FC and FS lines to intersect, i.e., in order to satisfy both FC and FS conditions. This is in line with the known behavior of DH functionals, which typically carry a larger percentage of HF exchange (often ∼50%).^26,27,32,98,108,109^

As a second way to constrain the number of free parameters in the above analysis, we set α_x_ to a fixed value, while we vary the values of γ_x_, γ_c_, and α_c_ independently. Results obtained using α_x_ = 0.2 are shown in Figure 2b-ii. We conclude that, if the range-separation parameters of the exchange and the correlation are allowed to be different, the FC and FS curves can still intersect. In this manner, use of a lower HF exchange fraction, which may be advantageous in terms of spin contamination,^32,110−114^ can be achieved. A higher fraction of α_x_ can still be used equally well: see section VI.B in the Supporting Information for a comparison of the tuning procedure of the DH-RSxc-ii functional with α_x_ = 0.2 and α_x_ = 0.5.

**Figure 3:**
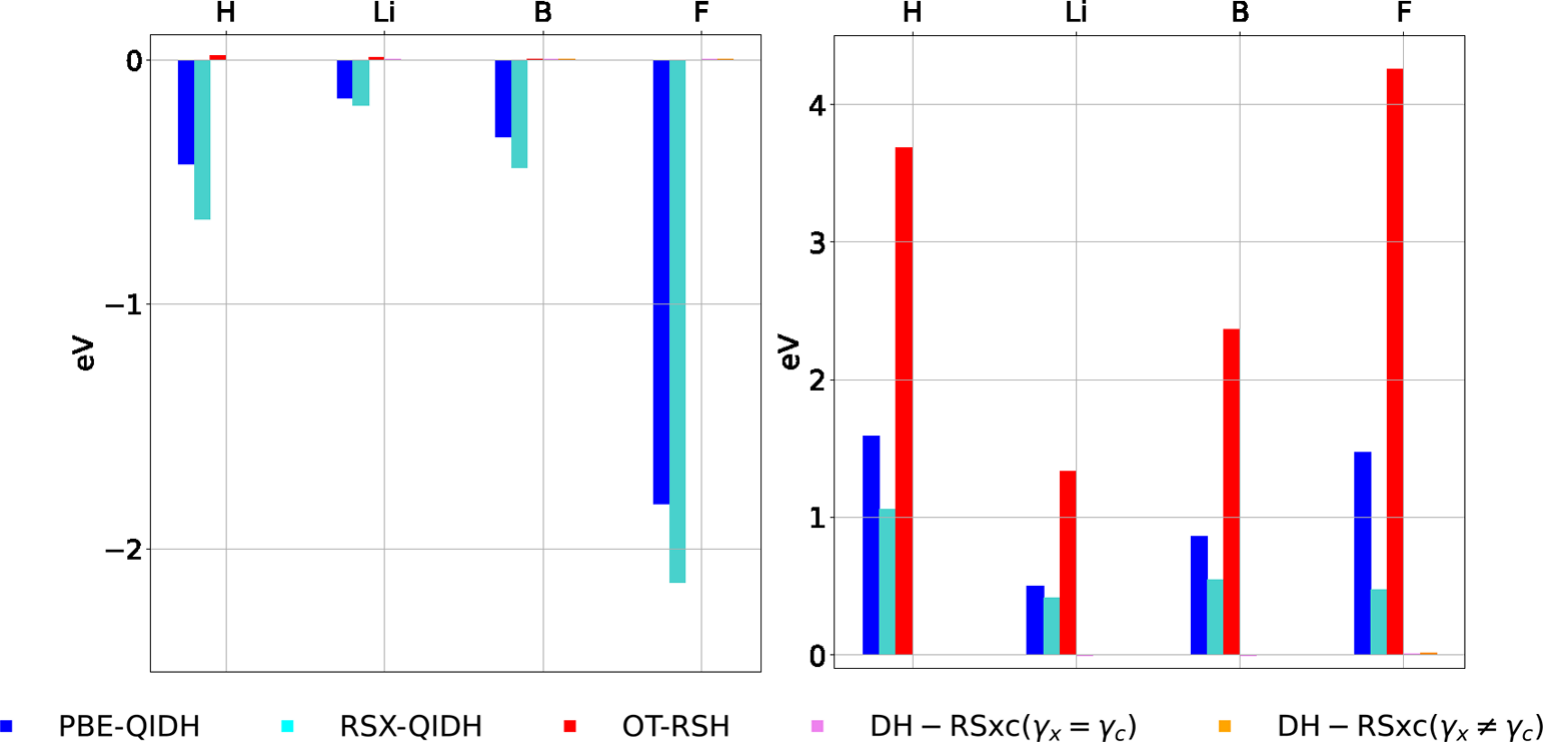
Middle point (left) FC and (right) FS errors for H, Li, B, and F atoms calculated with various PBE-based functionals: PBE-QIDH, a nontuned global double hybrid (blue ■); RSX-QIDH, a nontuned DH-RSx functional (cyan ■); OT-RSH (red ■) with α_x_ = 0.2; DH-RSxc (magenta ■) with γ_x_ = γ_c_ and α_c_ = 0.2; and DH-RSxc (orange ■) with α_x_ = 0.5 and α_c_ = 0.2. The parameters of the latter two functionals were determined from the intersection points of the middle point FC and FS contour lines, as shown in Figure 2b-i and Figure VI.3 of the Supporting Information, respectively, for the Li atom. Some of the functionals exhibit errors too small to see. (See section VII in the Supporting Information for the values of the errors and the parameters of the RSH functionals.)

To illustrate the relative merit of our approach, we compare in Figure 3 the “middle point” FC and FS errors for the H, Li, B, and F atoms, calculated with various PBE-based functionals: the nontuned PBE-QIDH^115^ functional (a global DH), the nontuned RSX-QIDH (a DH-RSx functional), (single) OT-RSH, and tuned OT-DH-RSxc with γ_x_ = γ_c_ and γ_x_ ≠ γ_c_. For the latter, we use α_x_ = 0.5, in order to facilitate the comparison with nontuned DH functionals that include a similar fraction of exact exchange. (See section VII in the Supporting Information for the parameters of each functional and exact numbers for the errors and section VIII for a respective comparison with BLYP-based DH functionals: B2PLYP, a global DH functional, and ωB2PLYP, a DH-RSx functional.) We observe that the PBE-QIDH and RSX-QIDH functionals show significant FC and FS errors for all atoms, OT-RSH shows very small FC errors but very high FS errors, but tuned DH-RSxc functionals maintain the low FC errors while also exhibiting low FS errors.

**Figure 4:**
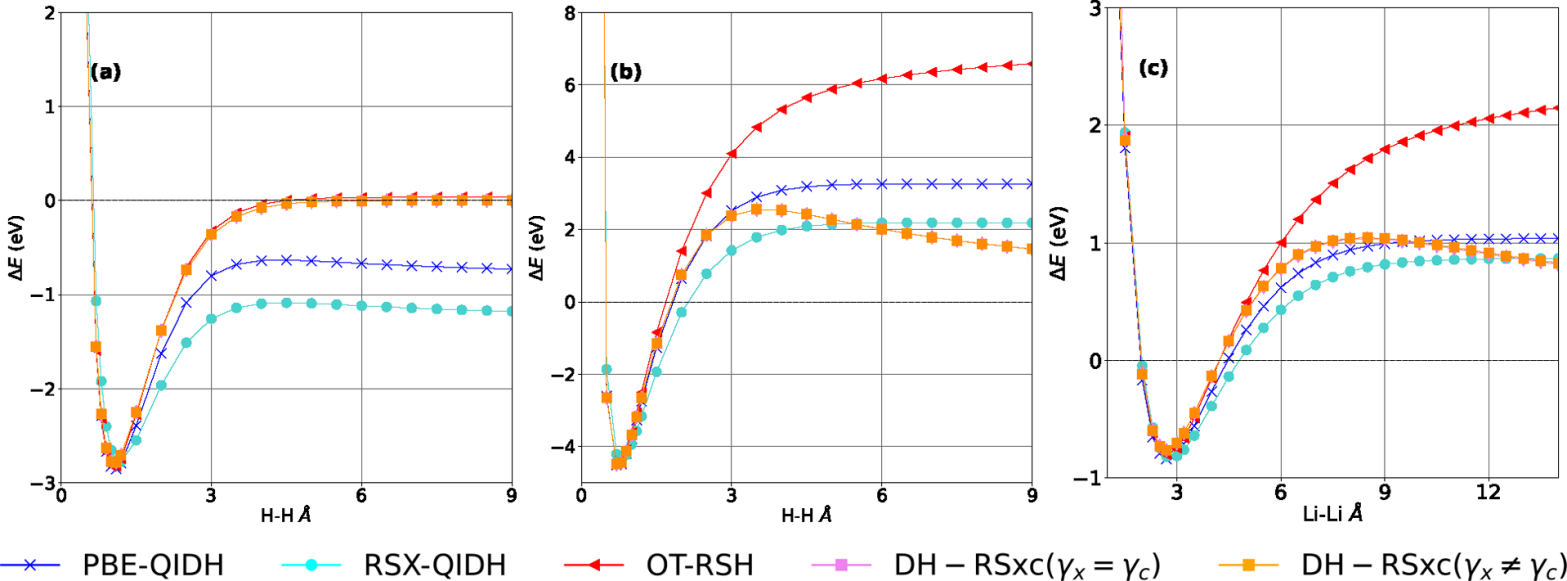
Dissociation curves of H_2_^+^, H_2_, and Li_2_, calculated with the same functionals used in Figure 3, namely, PBE-QIDH (−×−), RSX-QIDH (−●−), OT-RSH (−◀−), DH-RSxc-I (magenta −■−), and DH-RSxc-II (orange −■−).

Low FC and FS errors are important because we expect them to improve various predictions even for the parent integer electron case and related systems.^61^ Here, we test this by using the DH-RSxc functionals to compute the dissociation curves of diatomic molecules, which are known to be strongly affected by FC and FS errors^61,75^ and are a common and strong test case for new methods.^33,61,62,78,106,114,116−121^Figure 4 shows the obtained dissociation curves for the H_2_^+^, H_2_, and Li_2_ molecules, calculated with the same set of functionals used in Figure 3 (see section IX in Supporting Information for dissociation curves of the same systems, obtained with the DH-RSxc-ii functional but with a reduced fraction of exact exchange). In all calculations shown in Figure 4, the spin state was purposefully kept constant throughout the dissociation curve, to avoid improvements in energy owing to symmetry breaking.^22,122,123^ We note that the H_2_^+^ molecule is obviously a one-electron system; therefore, full HF exchange and zero correlation provide the exact result. OT-RSH and the two DH-RSxc functionals are accurate, in agreement with the fact that they exhibit low FC errors, but PBE-QIDH and RSX-QIDH both converge to a wrong dissociation limit. For H_2_ and Li_2_ atoms, correlation is nonzero and therefore the FS error becomes relevant. OT-RSH exhibits a higher middle point FS error (see Figure 3) for the H and Li atoms, when compared to the PBE-QIDH and RSX-QIDH functionals. This becomes apparent in the dissociation curves of the H_2_ and Li_2_ molecules, for which OT-RSH performs significantly worse than either the PBE-QIDH or the RSX-QIDH functional. Both of the DH-RSxc schemes perform significantly better than OT-RSH, but even though they tend to reach the correct asymptotic limit of zero at very large distances, they both exhibit a nonphysical maximum after equilibrium. This is not unprecedented: a similar feature appears in an MP2/DCPT2 calculation^75,124^ and has also been found by using the random phase approximation (RPA) method^62,125,126^ and a recent machine-learned functional.^100^ Clearly, a more subtle treatment of correlation is needed to eliminate this error. For example, Becke^127,128^ showed that variational optimization of fractional occupations improves the dissociation curve.

## Conclusions

In conclusion, we studied the optimal tuning of the free parameters in range-separated double hybrid functionals, based on enforcing two exact conditions: piecewise linearity and spin constancy. We found that introducing the range separation in both the exchange and correlation terms allowed for the minimization of both fractional charge and fractional spin errors for singlet atoms. The optimal set of parameters was found to be system specific, underscoring the importance of the tuning procedure. The performance of the resulting optimally tuned functionals for the dissociation curves of diatomic molecules has been tested. We found that they recover the correct dissociation curve for the one-electron system, H_2_^+^, and improve the dissociation curves of many-electron molecules such as H_2_ and Li_2_, but they also yield a nonphysical maximum and only tend to the correct dissociation limit at very large distances.

## Supporting Information Available

Range-separated MP2 and DCPT2; comparison of conventional and renormalized finite-temperature MP2 and DCPT2 expressions; short-range xc functionals; FC and FS errors of global double hybrid, DH-RSx, and DH-RSxc functionals; parameters and numerical values for Figures 3 and 4; bar plots and dissociation curves with BLYP-based DH functionals; and dissociation curves with DH-RSxc-ii and two different fractions of exact exchange (PDF)
