# Supplementary material for: Optimal Tuning Perspective of Range-Separated Double Hybrid Functionals
Source: J Chem Theory Comput. 2022 Apr 2;18(4):2331–40. doi: 10.1021/acs.jctc.2c00082 (PMC9009176; doi:10.1021/acs.jctc.2c00082)
Supplement: Supplementary file 1 — ct2c00082_si_001.pdf [file ct2c00082_si_001.pdf]

Supporting Information

An optimal tuning perspective of range-separated double hybrid functionals

Georgia Prokopiou[1], *Michal Hartstein*[1], *Niranjan Govind*[2] *and Leeor Kronik*[1,*]

[1]*Department of Molecular Chemistry and Materials Science, Weizmann Institute of Science, Rehovoth 76100, Israel*

[2]*Physical and Computational Sciences Directorate, Pacific Northwest National Laboratory, Richland, Washington 99352, USA*

CONTENTS

I. Range-separated MP2 and DCPT2 3

II. Comparison of conventional and renormalized finite-temperature MP2 and DCPT2 expressions 5

III. Short-Range xc functionals 6

IV. FC and FS errors of global double-hybrid functionals 9

V. FC and FS errors with the DH-RSx functionals 9

VI. FC and FS errors with the DH-RSxc functionals 11

A. Individual contour plots 11

B. Effect of the change of the fraction of the exact exchange in the DH-RSxc-ii functional 13

VII. Parameters and numerical values for figs 3, 4 14

VIII. Bar plots and dissociation curves with BLYP-based DH dunctionals 15

IX. Dissociation curves with DH-RSxc-ii and two different fractions of exact exchange 16

References 16

I. RANGE-SEPARATED MP2 AND DCPT2

We summarize the proof presented by Kalai and Toulouse,[1] where the following identity was used:

$$\omega_{ee}(r_{12}) = \underbrace{\left[\omega_{ee}^{\mathrm{LR}}(r_{12}) + \lambda\omega_{ee}^{\mathrm{SR}}(r_{12})\right]}_{\mathrm{MP2}} + \underbrace{(1-\lambda)\omega_{ee}^{\mathrm{SR}}(r_{12})}_{\mathrm{DFT}} \tag{I.1}$$

where $\omega_{ee}(r_{12}) = \frac{1}{r_{12}}$, $\omega_{ee}^{\mathrm{LR}}(r_{12}) = \frac{\mathrm{erf}(\mu r_{12})}{r_{12}}$, $\omega_{ee}^{\mathrm{SR}}(r_{12}) = \frac{\mathrm{erfc}(\mu r_{12})}{r_{12}}$, and μ is the range-separation parameter. Here we add $(\alpha+\beta)$ and α as coefficient for the LR and SR terms respectively, which is a more general case. Eq. (I.1) is retrieved by setting $\alpha+\beta = 1$ and $\alpha = \lambda$. Assuming real i, j, a, b orbitals, the numerator of the MP2 term is calculated as follows:

$$\begin{aligned}
&|\langle ij||ab\rangle|^2 = |\langle ij\,|\omega_{ee}(r_{12})|\,ab\rangle - \langle ij\,|\omega_{ee}(r_{12})|\,ba\rangle|^2 = \\
&\left|\left\langle ij\,\middle|(\alpha+\beta)\,\omega_{\mathrm{ee}}^{\mathrm{LR}} + \alpha\omega_{\mathrm{ee}}^{\mathrm{SR}}\middle|\,ab\right\rangle - \left\langle ij\,\middle|(\alpha+\beta)\,\omega_{\mathrm{ee}}^{\mathrm{LR}} + \alpha\omega_{\mathrm{ee}}^{\mathrm{SR}}\middle|\,ba\right\rangle\right|^2 = \\
&\left\langle ij\,\middle|(\alpha+\beta)\,\omega_{\mathrm{ee}}^{\mathrm{LR}} + \alpha\omega_{\mathrm{ee}}^{\mathrm{SR}}\middle|\,ab\right\rangle^2 + \left\langle ij\,\middle|(\alpha+\beta)\,\omega_{\mathrm{ee}}^{\mathrm{LR}} + \alpha\omega_{\mathrm{ee}}^{\mathrm{SR}}\middle|\,ba\right\rangle^2 \\
&-2\left\langle ij\,\middle|(\alpha+\beta)\,\omega_{\mathrm{ee}}^{\mathrm{LR}} + \alpha\omega_{\mathrm{ee}}^{\mathrm{SR}}\middle|\,ab\right\rangle\left\langle ij\,\middle|(\alpha+\beta)\,\omega_{\mathrm{ee}}^{\mathrm{LR}} + \alpha\omega_{\mathrm{ee}}^{\mathrm{SR}}\middle|\,ba\right\rangle
\end{aligned} \tag{I.2}$$

The first term of Eq. (I.2) is expanded as:

$$\begin{aligned}
&\left\langle ij\,\middle|(\alpha+\beta)\,\omega_{\mathrm{ee}}^{\mathrm{LR}} + \alpha\omega_{\mathrm{ee}}^{\mathrm{SR}}\middle|\,ab\right\rangle^2 = (\alpha+\beta)^2\,\langle ij|ab\rangle_{\mathrm{LR}}^2 + \alpha^2\,\langle ij|ab\rangle_{\mathrm{SR}}^2 \\
&+2\,(\alpha+\beta)\,\alpha\,\langle ij|ab\rangle_{\mathrm{LR}}\,\langle ij|ab\rangle_{\mathrm{SR}}\,,
\end{aligned} \tag{I.3}$$

where $\langle ij|ab\rangle_{\mathrm{LR}} = \langle ij|\omega_{\mathrm{ee}}^{\mathrm{LR}}|ab\rangle$ and $\langle ij|ab\rangle_{\mathrm{SR}} = \langle ij|\omega_{\mathrm{ee}}^{\mathrm{SR}}|ab\rangle$

The second term of Eq. (I.2) is expanded as:

$$\begin{aligned}
&\left\langle ij\,\middle|(\alpha+\beta)\,\omega_{\mathrm{ee}}^{\mathrm{LR}} + \alpha\omega_{\mathrm{ee}}^{\mathrm{SR}}\middle|\,ba\right\rangle^2 = (\alpha+\beta)^2\,\langle ij|ba\rangle_{\mathrm{LR}}^2 + \alpha^2\,\langle ij|ba\rangle_{\mathrm{SR}}^2 \\
&+2\,(\alpha+\beta)\,\alpha\,\langle ij|ba\rangle_{\mathrm{LR}}\,\langle ij|ba\rangle_{\mathrm{SR}}\,,
\end{aligned} \tag{I.4}$$

And the third term of Eq. (I.2) is expanded as:

$$\begin{aligned}
&-2\left\langle ij\,\middle|(\alpha+\beta)\,\omega_{\mathrm{ee}}^{\mathrm{LR}} + \alpha\omega_{\mathrm{ee}}^{\mathrm{SR}}\middle|\,ab\right\rangle\left\langle ij\,\middle|(\alpha+\beta)\,\omega_{\mathrm{ee}}^{\mathrm{LR}} + \alpha\omega_{\mathrm{ee}}^{\mathrm{SR}}\middle|\,ba\right\rangle, \\
&= -2\,(\alpha+\beta)^2\,\langle ij|ab\rangle_{\mathrm{LR}}\,\langle ij|ba\rangle_{\mathrm{LR}} - 2\alpha^2\,\langle ij|ab\rangle_{\mathrm{SR}}\,\langle ij|ba\rangle_{\mathrm{SR}} \\
&-2\,(\alpha+\beta)\,\alpha\,\langle ij|ab\rangle_{\mathrm{LR}}\,\langle ij|ba\rangle_{\mathrm{SR}} - 2\,(\alpha+\beta)\,\alpha\,\langle ij|ab\rangle_{\mathrm{SR}}\,\langle ij|ba\rangle_{\mathrm{LR}}
\end{aligned} \tag{I.5}$$

Using the following identities:

$$[(\alpha+\beta)\,\langle ij|ab\rangle_{\mathrm{LR}} - (\alpha+\beta)\,\langle ij|ba\rangle_{\mathrm{LR}}]^2 = (\alpha+\beta)^2\,\langle ij||ab\rangle_{\mathrm{LR}}^2 \tag{I.6}$$

$$\left[\alpha \langle ij|ab\rangle_{\mathrm{SR}} - \alpha \langle ij|ba\rangle_{\mathrm{SR}}\right]^2 = \alpha^2 \langle ij||ab\rangle^2_{\mathrm{SR}} \tag{I.7}$$

$$\begin{aligned}
&-2(\alpha+\beta)\,\alpha\,\langle ij|ba\rangle_{\mathrm{SR}}\left[\langle ij|ab\rangle_{\mathrm{LR}} - \langle ij|ba\rangle_{\mathrm{LR}}\right] + 2(\alpha+\beta)\,\alpha\,\langle ij|ab\rangle_{\mathrm{SR}}\left[\langle ij|ab\rangle_{\mathrm{LR}} - \langle ij|ba\rangle_{\mathrm{LR}}\right] \\
&= +2(\alpha+\beta)\,\alpha\,\langle ij||ab\rangle_{\mathrm{SR}}\,\langle ij||ab\rangle_{\mathrm{LR}}
\end{aligned} \tag{I.8}$$

Eq. (I.2) is simplified to:

$$\begin{aligned}
&\left|\left\langle ij\left|(\alpha+\beta)\,\omega_{\mathrm{ee}}^{\mathrm{LR}} + \alpha\omega_{\mathrm{ee}}^{\mathrm{SR}}\right| ab\right\rangle - \left\langle ij\left|(\alpha+\beta)\,\omega_{\mathrm{ee}}^{\mathrm{LR}} + \alpha\omega_{\mathrm{ee}}^{\mathrm{SR}}\right| ba\right\rangle\right|^2 = \\
&(\alpha+\beta)^2\langle ij||ab\rangle^2_{\mathrm{LR}} + \alpha^2\langle ij||ab\rangle^2_{\mathrm{SR}} + 2(\alpha+\beta)\,\alpha\,\langle ij||ab\rangle_{\mathrm{SR}}\,\langle ij||ab\rangle_{\mathrm{LR}}
\end{aligned} \tag{I.9}$$

Therefore, the RS-MP2 energy is calculated as:

$$\begin{aligned}
E_{\mathrm{c}}^{\mathrm{RS-MP2}} &= -\sum_{i<j}^{\mathrm{occ}}\sum_{a<b}^{\mathrm{virt}} \frac{\left|\left\langle ij\left|(\alpha+\beta)\,\omega_{\mathrm{ee}}^{\mathrm{LR}} + \alpha\omega_{\mathrm{ee}}^{\mathrm{SR}}\right| ab\right\rangle - \left\langle ij\left|(\alpha+\beta)\,\omega_{\mathrm{ee}}^{\mathrm{LR}} + \alpha\omega_{\mathrm{ee}}^{\mathrm{SR}}\right| ba\right\rangle\right|^2}{\epsilon_a + \epsilon_b - \epsilon_i - \epsilon_j} \\
&= -\sum_{i<j}^{\mathrm{occ}}\sum_{a<b}^{\mathrm{virt}} \frac{(\alpha+\beta)^2\langle ij||ab\rangle^2_{\mathrm{LR}} + \alpha^2\langle ij||ab\rangle^2_{\mathrm{SR}} + 2(\alpha+\beta)\,\alpha\,\langle ij||ab\rangle_{\mathrm{SR}}\,\langle ij||ab\rangle_{\mathrm{LR}}}{\epsilon_a + \epsilon_b - \epsilon_i - \epsilon_j}
\end{aligned} \tag{I.10}$$

Similarly, the RS-DCPT2 is given by:

$$\begin{aligned}
E_{\mathrm{c}}^{\mathrm{RS-DCPT2}} = \frac{1}{8}\sum_{ij}^{\mathrm{occ}}\sum_{ab}^{\mathrm{virt}}\Big[D_{abij} \\
-\sqrt{(D_{abij})^2 + 4(\alpha_{\mathrm{c}}+\beta_{\mathrm{c}})^2\langle ij||ab\rangle^2_{\mathrm{LR},\gamma_{\mathrm{c}}} + 4\alpha_{\mathrm{c}}^2\langle ij||ab\rangle^2_{\mathrm{SR},\gamma_{\mathrm{c}}} + 8(\alpha_{\mathrm{c}}+\beta_{\mathrm{c}})\,\alpha_{\mathrm{c}}\,\langle ij||ab\rangle_{\mathrm{SR},\gamma_{\mathrm{c}}}\langle ij||ab\rangle_{\mathrm{LR},\gamma_{\mathrm{c}}}}\Big]
\end{aligned} \tag{I.11}$$

In Fig. I.1 we plot the RS-MP2 and RS-DCPT2 energies for the Li atom, evaluated using the Hartree-Fock obritals and eigenvalues, with respect to the range-separation parameter γ_{c}. Here and throughout the rest of the SI we change the notation from (μ, α, β) to $(\gamma_{\mathrm{c}}, \alpha_{\mathrm{c}}, \beta_{\mathrm{c}})$ in order to distinguish between the range-separation parameters for the exchange and the correlation terms. In the first plot, where $\alpha_{\mathrm{c}} = 0.0$ and $\beta_{\mathrm{c}} = 1.0$, only the LR term in Eq. (I.10) and Eq. (I.11) survives. In the second plot where $\alpha_{\mathrm{c}} = 0.2$ and $\beta_{\mathrm{c}} = 0.8$, all the terms contribute. For both cases, the RS-MP2/DCPT2 energies approach the full-range (FR)-MP2/DCPT2 energy as γ_{c} increases. In the third plot, where $\alpha_{\mathrm{c}} = 1.0$ and $\beta_{\mathrm{c}} = 0.0$, the FR-MP2/DCPT2 energy is recovered for all the values of γ_{c}.

We note that even though DCPT2 treats the divergence of MP2 when $D_{abij} \to 0$ DCPT2 still diverges when $< ij||ab > \to 0$ and $D_{abij} \to -\infty$.[2, 3] These divergent terms arise when fractional occupations are used (see Eq. (14) in main text), because both summations include all molecular orbitals, which allows D_{abij} to be negative. In our implementation, we ignore all terms with $D_{abij} < 0$, which correspond to de-excitations.

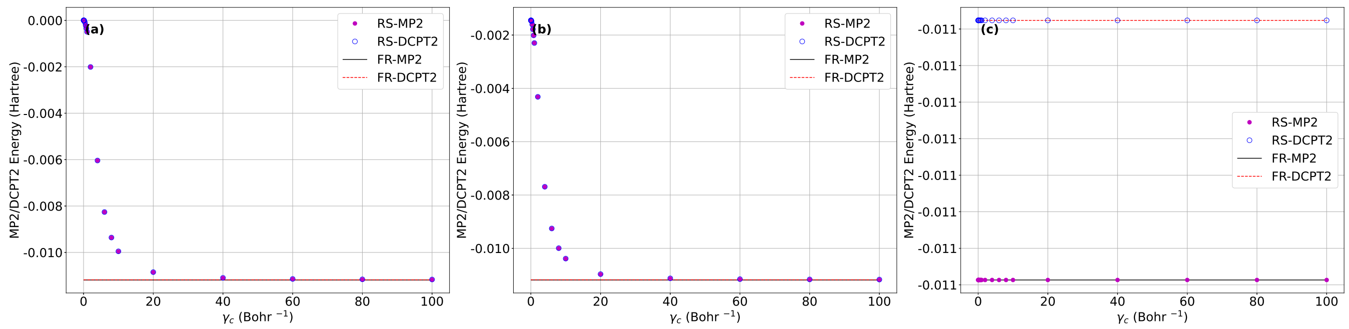

FIG. I.1. RS-MP2/DCPT2 and FR-MP2/DCPT2 energies (in Hartree), for the Li atom, evaluated based on a Hartree-Fock calculation, with respect to the range-separation parameter γ_c, using $\alpha_c + \beta_c = 1.0$ and **(a)** $\alpha_c = 0.0$, **(b)** $\alpha_c = 0.2$, **(c)** $\alpha_c = 1.0$.

II. COMPARISON OF CONVENTIONAL AND RENORMALIZED FINITE-TEMPERATURE MP2 AND DCPT2 EXPRESSIONS

Here we discuss the conventional and the renormalized MP2, as well as the respective DCPT2 expressions. The conventional finite-temperature MP2 and DCPT2 expressions are:

$$E_c^{\mathrm{MP2}} = \frac{1}{4} \sum_{ij}^{\mathrm{all}} \sum_{ab}^{\mathrm{all}} \delta_i \delta_j \frac{\langle ij||ab \rangle^2}{\epsilon_i + \epsilon_j - \epsilon_a - \epsilon_b} (1-\delta_a)(1-\delta_b), \tag{II.1}$$

and

$$E_c^{\mathrm{DCPT2}} = \frac{1}{8} \sum_{ij}^{\mathrm{all}} \sum_{ab}^{\mathrm{all}} D_{abij} - \sqrt{(D_{abij})^2 + 4\delta_i \delta_j \langle ij||ab \rangle^2 (1-\delta_a)(1-\delta_b)}, \tag{II.2}$$

where $D_{abij} = \epsilon_a + \epsilon_b - \epsilon_i - \epsilon_j$. Hirata *et al.* recently introduced [4, 5] a renormalized expression for the MP2 correlation term, which recovers the correct zero-temperature limit for metals:

$$E_c^{\mathrm{renorm-MP2}} = \frac{1}{4} \sum_{ij}^{\mathrm{all}} \sum_{ab}^{\mathrm{all}} \delta_i \delta_j \frac{\langle ij||ab \rangle^2}{\delta_i \epsilon_i + \delta_j \epsilon_j - (1-\delta_a)\epsilon_a - (1-\delta_b)\epsilon_b} (1-\delta_a)(1-\delta_b) \tag{II.3}$$

Based on the above equation, the DCPT2 term becomes:

$$E_c^{\mathrm{renorm-DCPT2}} = \frac{1}{8} \sum_{ij}^{\mathrm{all}} \sum_{ab}^{\mathrm{all}} D_{abij}^{\mathrm{renorm}} - \sqrt{(D_{abij}^{\mathrm{renorm}})^2 + 4\delta_i \delta_j \langle ij||ab \rangle^2 (1-\delta_a)(1-\delta_b)} \tag{II.4}$$

where $D_{abij}^{\mathrm{renorm}} = (1-\delta_a)\epsilon_a + (1-\delta_b)\epsilon_b - \delta_i \epsilon_i - \delta_j \epsilon_j$.

In Fig. II.1 we plot the FC and FS curves for the Li atom using Eq. (II.1) - Eq. (II.4).

In the fractional charge case, the MP2 and the DCPT2 terms coincide for both the conventional and the renormalized terms, as expected for a non-degenerate system. The renormalized curve deviates from the conventional one. While this deviation is small ($\sim 0.5\mathrm{e}{-5}$ Hartree) it is comparable to the individual values of the curves, therefore it is not

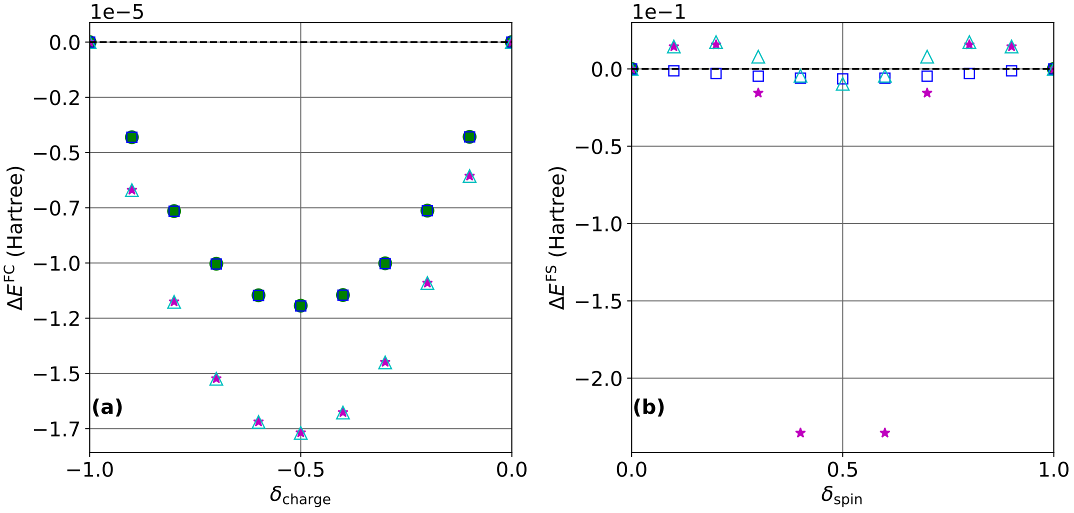

FIG. II.1. **(a)**: FC error curves (Eq. (10) in main text) and **(b)**: FS error curves (Eq. (11) in main text) in Hartree, for the Li atom, calculated with the Hartree-Fock+MP2/DCPT2 using the conventional MP2 (●), the renormalized MP2 (★), the conventional DCPT2 (□), and the renormalized DCPT2 (△) expressions.

negligible [6]. In the FS case, the conventional MP2 curve diverges in all non-integer points. The renormalized MP2 curve yields reasonable values for the first and last three points, but as we approach $\delta_{\text{spin}} = 0.5$ it also diverges. Similarly to the FC curve, the two DCPT2 terms yield different FS curves and again this difference is not negligible due to the small values of each FS curve. Based on this analysis, it is evident that the tuned parameters are expected to be somewhat different when the renormalized expressions are used. However, this will not affect significantly the dissociation curves, because the renormalized and the conventional expressions are identical for integer occupations.

III. SHORT-RANGE XC FUNCTIONALS

Here we present the basic equations of the range-separated LDA and PBE xc functionals, for a closed -shell system, following the work of Toulouse *et al.* [7, 8], Paziani *et al.* [9] and Goll *et al.* [10]

The SR-PBE exchange energy is [10]:

$$E_{\text{x}}^{\text{PBE-SR}} = \int d\mathbf{r}\;\; n(\mathbf{r})\epsilon_{\text{x}}^{\text{SR-LDA}}\left(\mu, n(\mathbf{r})\right) F_{\text{x}}^{\text{PBE-SR}}\left(s, \mu\right) \tag{III.1}$$

where $s = \frac{|\nabla n(\mathbf{r})|}{2k_{\text{F}} n(\mathbf{r})}$ with $k_{\text{F}} = \left(3\pi^2 n(\mathbf{r})\right)^{\frac{1}{3}}$ and μ is the range-separation parameter. The enhancement factor, $F_{\text{x}}(s, \mu)$, is defined as [10]:

$$F_{\text{x}}(s, \mu) = 1 + \kappa - \frac{\kappa}{1 + b(\mu)s^2/\kappa} \tag{III.2}$$

with $\kappa = 0.840$, $\tilde{\mu} = \frac{\mu}{2k_\mathrm{F}}$ and the gradient coefficient is defined as [10]:

$$b(\mu) = \frac{b^{\mathrm{PBE}}}{b^{\mathrm{T}}(0)} b^{\mathrm{T}}(\tilde{\mu}) e^{-\alpha_\mathrm{x}\tilde{\mu}^2} \tag{III.3}$$

with $\alpha_\mathrm{x} = 19.0$, $b^{\mathrm{PBE}} = 0.21951$, and $b^{\mathrm{T}}(\tilde{\mu})$ being a gradient coefficient defined by Toulouse *et al.* [8] as:

$$b^{\mathrm{T}}(\tilde{\mu}) = \frac{-c_1 + c_2 e^{1/(4\tilde{\mu}^2)}}{c_3 + 54 c_4 e^{1/(4\tilde{\mu}^2)}} \tag{III.4}$$

with

$$\begin{aligned}
c_1 &= 1 + 22\tilde{\mu}^2 + 144\tilde{\mu}^4 \\
c_2 &= 2\tilde{\mu}^2\left(-7 + 72\tilde{\mu}^2\right) \\
c_3 &= -864\tilde{\mu}^4\left(-1 + 2\tilde{\mu}^2\right) \\
c_4 &= \tilde{\mu}^2\left[-3 - 24\tilde{\mu}^2 + 32\tilde{\mu}^4 + 8\tilde{\mu}\sqrt{\pi}\mathrm{erf}\left(\frac{1}{2\tilde{\mu}}\right)\right]
\end{aligned} \tag{III.5}$$

The short-range LDA exchange energy per particle, $\epsilon_\mathrm{x}^{\mathrm{SR-LDA}}(\mu, n(\mathbf{r}))$ is defined as [7, 9]:

$$\epsilon_\mathrm{x}^{\mathrm{SR-LDA}}(\mu, n(\mathbf{r})) = \epsilon_\mathrm{x}^{\mathrm{LDA}}(n(\mathbf{r})) - \epsilon_\mathrm{x}^{\mathrm{LR-LDA}}(\mu, n(\mathbf{r})) \tag{III.6}$$

where $\epsilon_\mathrm{x}^{\mathrm{LDA}} = A_\mathrm{x} n(\mathbf{r})^{1/3} = -\frac{3}{4}\left(\frac{3n(\mathbf{r})}{\pi}\right)^{1/3}$ is the LDA exchange energy per particle and $\epsilon_\mathrm{x}^{\mathrm{LR-LDA}}$ is the long-range LDA exchange energy per particle defined as [7, 9]:

$$\epsilon_\mathrm{x}^{\mathrm{LR-LDA}} = -\frac{\mu}{\pi}\left[\left(2y - 4y^3\right)e^{-1/(4y^2)} - 3y + 4y^3 + \sqrt{\pi}\mathrm{erf}\left(\frac{1}{2y}\right)\right] \tag{III.7}$$

with $y = \frac{\mu}{2k_\mathrm{F}}$.

The SR-PBE correlation energy is [10]:

$$E_\mathrm{c}^{\mathrm{SR-PBE}} = \int d\mathbf{r}\;\; n(\mathbf{r})\left[\epsilon_\mathrm{c}^{\mathrm{SR-LDA}}(\mu, n(\mathbf{r})) + H(\mu, n(\mathbf{r}), t)\right] \tag{III.8}$$

where

$$H(\mu, n(\mathbf{r}), t) = \gamma \ln\left\{1 + \frac{\beta(\mu)\mathrm{t}^2}{\gamma}\left[\frac{1 + \mathrm{A}\mathrm{t}^2}{1 + \mathrm{A}\mathrm{t}^2 + \mathrm{A}^2\mathrm{t}^4}\right]\right\} \tag{III.9}$$

where

$$A = \frac{\beta(\mu)}{\gamma\left(e^{\left(-\epsilon_\mathrm{c}^{\mathrm{SR-LDA}}/\gamma\right)} - 1\right)} \tag{III.10}$$

with $t = \frac{|\nabla n(\mathbf{r})|}{2k_\mathrm{s} n(\mathbf{r})}$, $k_\mathrm{s} = \sqrt{\frac{4k_\mathrm{F}}{\pi}}$ and $\gamma = 0.031091$. The gradient coefficient, $\beta(\mu)$, is defined as [10]:

$$\beta(\mu) = \beta^{\mathrm{PBE}}\left(\frac{\epsilon_\mathrm{c}^{\mathrm{SR-LDA}}(\mu, n(\mathbf{r}))}{\epsilon_\mathrm{c}^{\mathrm{SR-LDA}}(0, n(\mathbf{r}))}\right)^{\alpha_\mathrm{c}} \tag{III.11}$$

with $\beta^{\mathrm{PBE}} = 0.066725$, $\alpha_{\mathrm{c}} = 2.83$, and the correlation energy per particle, $\epsilon_{\mathrm{c}}^{\mathrm{SR-LDA}}(\mu, n(\mathbf{r}))$, defined as in Refs. [7, 9].

We implemented the range-separated xc LDA and PBE functionals presented in Ref. [9] and Refs. [8, 10]. We tested the implementation for a few systems. A comparison of the total energy of He atom obtained from our implementation in `NWchem` and `MOLPRO v.2019.2` [11, 12], as a function of the range-separation parameter, γ, is shown in Fig. III.1. The PBE and LDA functionals are recovered with the SR-PBE functional with $\gamma = 0$ and $\gamma \to \infty$, respectively, as expected.

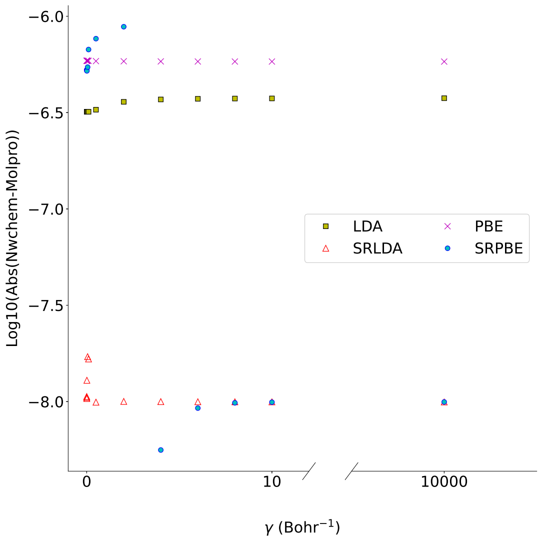

FIG. III.1. The absolute value of the difference between the **`NWchem`** and **`Molpro`** codes (in logarithmic scale) for the He atom (with cc-pvtz basis set), calculated with LDA, SR-LDA, PBE, SR-PBE + LR-HF functional

IV. FC AND FS ERRORS OF GLOBAL DOUBLE-HYBRID FUNCTIONALS

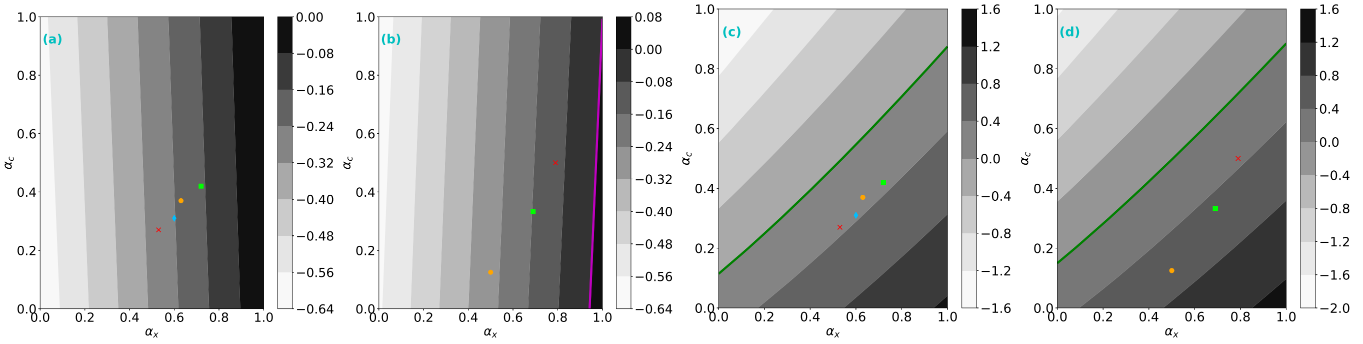

FIG. IV.1. **(a, b)**: FC errors (Eq. (10) in main text) for $\delta = -0.5$, **(c, d)**: FS errors (Eq. (11) in main text) for $\delta = 0.5$ in eV, for the Li atom, as a function of α_x (horizontal axis) and α_c (vertical axis). The associated total energies were calculated with the DH-RSx functional (Eq. (4) in main text). The Becke88 [13] exchange and the LYP [14] correlation functionals were used for **(a,c)** and the PBE exchange and correlation functionals [15] were used for **(b, d)**. Common global double-hybrid functionals, along with their $(\alpha_\mathrm{x}, \alpha_\mathrm{c})$ parameters are also shown in **(a, c)**: B2PLYP [16], ×, (0.53, 0.27); B2GP-PLYP [17], ●, (0.63, 0.37); B2K-PLYP [18], ■, (0.72, 0.42); B2T-PLYP [17], ◆, (0.60, 0.31) **(b, d)**: PBE0-2 [19], ×, (0.79, 0.50); PBE0-DH [20], ●, (0.50, 0.125); PBE-QIDH [21], ■, (0.69, 0.33). For each contour plot the contour line corresponding to 10^{-4} eV is shown with solid colored line.

Here we study the FC and FS errors of global double-hybrid (DH) functionals, given by:

$$E_\mathrm{xc}^\mathrm{DH} = \alpha_\mathrm{x} E_\mathrm{x}^\mathrm{Fock} + (1-\alpha_\mathrm{x})\, E_\mathrm{x}^\mathrm{DFA} + \alpha_\mathrm{c} E_\mathrm{c}^\mathrm{DCPT2} + (1-\alpha_\mathrm{c})\, E_\mathrm{c}^\mathrm{DFA}. \tag{IV.1}$$

In Fig. IV.1 we show the FC and FS errors as we vary the α_x and α_c coefficients of two commonly used global double-hybrid functionals, B2PLYP and PBE0-DH. Evidently, it is not possible to minimize both FC and FS errors for the Li atom with Eq. (IV.1).

V. FC AND FS ERRORS WITH THE DH-RSX FUNCTIONALS

In Fig. V.1 we present contour plots for the middle FC and FS errors as we vary α_x, α_c, and γ_x in the DH-RSx equation:

$$E_\mathrm{xc}^\mathrm{DH-RSx} = \alpha_\mathrm{x} E_{\mathrm{SR},\gamma_\mathrm{x}}^\mathrm{Fock} + (1-\alpha_\mathrm{x}) E_{\mathrm{SR},\gamma_\mathrm{x}}^\mathrm{PBE} + E_{\mathrm{LR},\gamma_\mathrm{x}}^\mathrm{Fock} + \alpha_\mathrm{c} E_\mathrm{c}^\mathrm{MP2/DCPT2} + (1-\alpha_\mathrm{c})\, E_\mathrm{c}^\mathrm{PBE} \tag{V.1}$$

In order to analyze the trends shown in Fig. V.1, we describe briefly the corresponding limits. The parameters α_x and γ_x only affect the exchange part. When $\alpha_\mathrm{x} = 0.0$ DFT exchange is only used in the SR and HF is only used in the

LR. As α_x increases, more HF exchange is added in the SR which makes the FC and the FS errors more positive. The separation of the inter-electronic distance into SR and LR occurs at roughly $1/\gamma_\mathrm{x}$ Bohr. Therefore, when $\gamma_\mathrm{x} = 0.0$ the SR terms dominate while they are suppressed as γ_x increases. As γ_x decreases, the SR becomes longer. Therefore more DFT exchange is added, which makes the FC and FS curves more convex.

The parameter α_c controls the mixing of DFT and MP2/DCPT2 correlation terms. As α_c increases, more MP2/DCPT2 is added, which makes the FC and FS curves more convex. At the same time, $\gamma_\mathrm{x}^\mathrm{opt}$ (see highlighted contour lines in Fig. V.1) increases for larger α_c, in order to reduce the FC and FS errors. Additionally, we observe that for constant $(\alpha_\mathrm{x}, \alpha_\mathrm{c})$, $\gamma_\mathrm{x}^\mathrm{FC,opt}$ is always larger than $\gamma_\mathrm{x}^\mathrm{FS,opt}$, which reflects the fact that a higher percentage of HF reduces the FC error, while it increases the FS error.

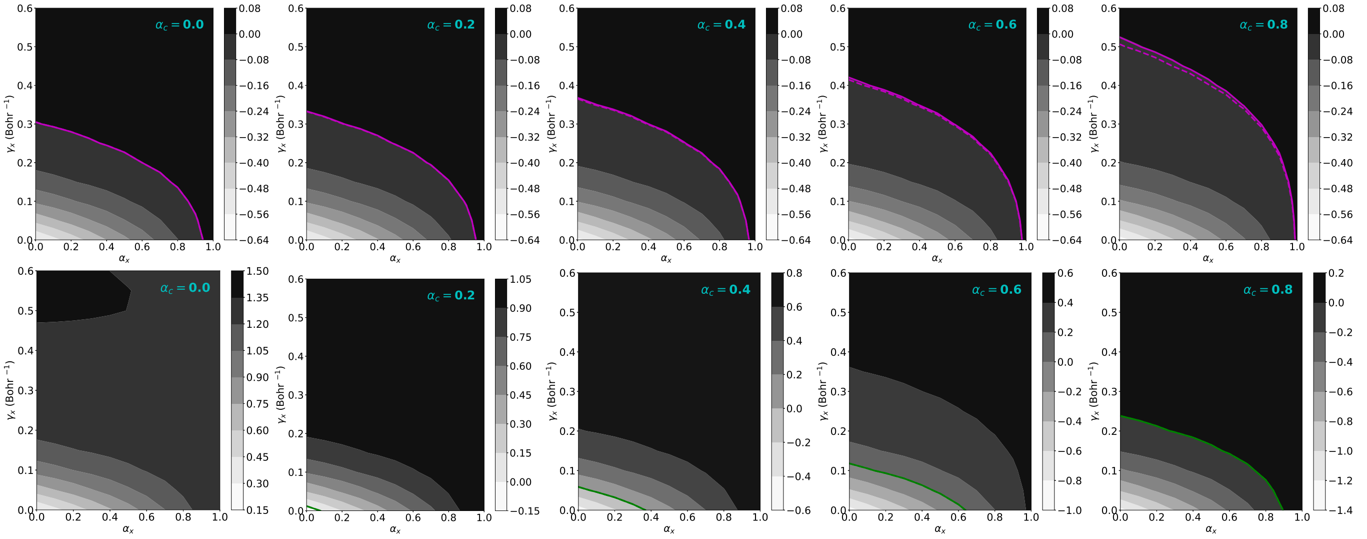

FIG. V.1. Li atom: **Upper panel**: FC errors (Eq. (10) in main text) for $\delta = -0.5$, **Lower panel**: FS errors (Eq. (11) in main text) for $\delta = 0.5$, in eV, as a function of α_x (horizontal axis), γ_x (vertical axis), and α_c (increasing from left ($\alpha_\mathrm{c} = 0.0$) to right ($\alpha_\mathrm{c} = 0.8$)). The associated total energies were calculated with the DH-RSx functional (Eq. (4) in main text). For each contour plot the contour line corresponding to 10^{-4} eV is shown with a solid colored line. The contour line based on the associated DFT total energies is also shown, with a dashed colored line.

VI. FC AND FS ERRORS WITH THE DH-RSXC FUNCTIONALS

A. Individual contour plots

Here we present contour plots for the middle FC and FS errors as we vary α_{x}, α_{c}, γ_{x}, and γ_{c} in the DH-RSxc equation:

$$E_{\mathrm{xc}}^{\mathrm{DH-RSxc}} = E_{\mathrm{x}}^{\mathrm{RSH}} + (1-\alpha_{\mathrm{c}})\,E_{\mathrm{SR},\gamma_{\mathrm{c}}}^{\mathrm{DFA}} + E_{\mathrm{c}}^{\mathrm{RS-MP2/DCPT2}} \tag{VI.1}$$

where the RS-MP2/DCPT2 terms are given in Eq. (I.10) and Eq. (I.11). Note that in the DH-RSxc scheme, the LR-MP2 component is "always on", *i.e.*, even with $\alpha_{\mathrm{c}} = 0.0$ there is MP2 correlation in the functional. This is in contrast to the previously discussed DH-RSx scheme where with $\alpha_{\mathrm{c}} = 0.0$ we recover a single hybrid functional. In Fig. VI.1 and Fig. VI.2 we show the contour plots for the DH-RSxc-I ($\gamma_{\mathrm{x}} = \gamma_{\mathrm{c}}$) and DHRSxc-II ($\alpha_{\mathrm{x}} = 0.2$) schemes, respectively. The effect of the α_{x} and γ_{x} on the FC and FS errors is the same as that described in the previous section. The α_{c} and γ_{c} parameters now control the range-separation of the correlation. With increasing α_{c} and γ_{c}, more MP2/DCPT2 correlation is included in the SR, which makes the FC and FS errors less positive (opposite to the effect of increasing α_{x} and γ_{x} which makes the errors more positive).

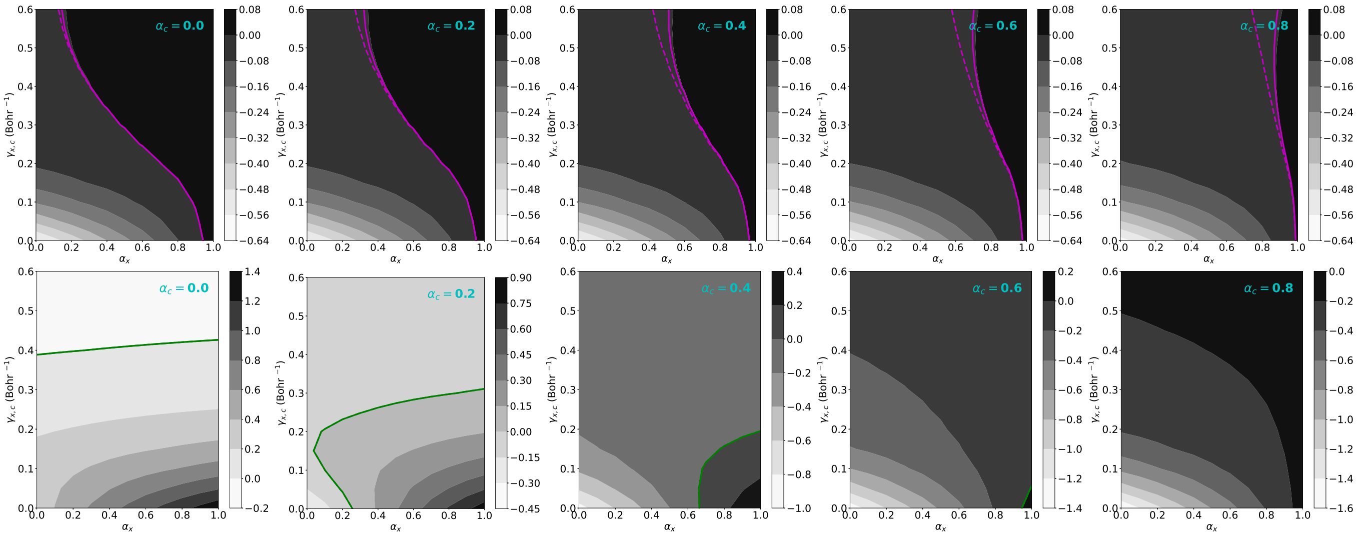

FIG. VI.1. Li atom: **Upper panel**: FC errors (Eq. (10) in main text) for $\delta = -0.5$, **Lower panel**: FS errors (Eq. (11) in main text) for $\delta = 0.5$, in eV, as a function of α_{x} (horizontal axis), $\gamma_{\mathrm{x,c}}$ (vertical axis) and α_{c} (increasing from left ($\alpha_{\mathrm{c}} = 0.0$) to right ($\alpha_{\mathrm{c}} = 0.8$)). The associated total energies were calculated with the DH-RSxc functional (Eq. (7) in main text). For each contour plot, the contour line corresponding to 10^{-4} eV is shown with a solid colored line. The contour line based on the associated DFT total energies is also shown with a dashed colored line.

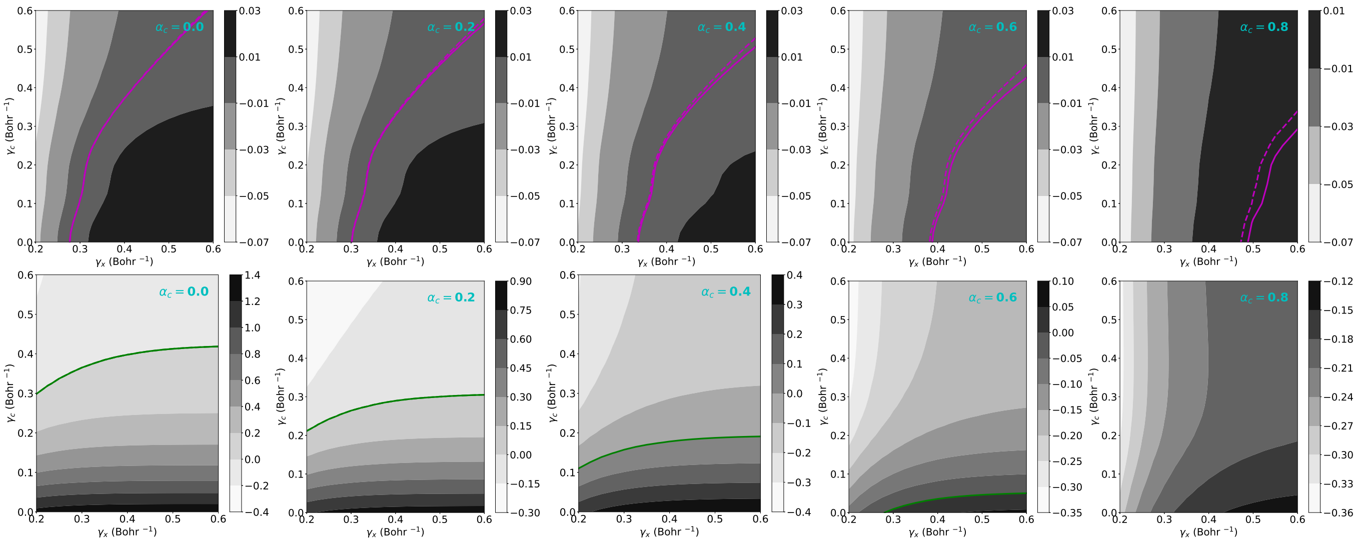

FIG. VI.2. Li atom: **Upper panel**: FC errors (Eq. (10) in main text) for $\delta = -0.5$, **Lower panel**: FS errors (Eq. (11) in main text) for $\delta = 0.5$, in eV as a function of $\gamma_{\rm x}$ (horizontal axis), $\gamma_{\rm c}$ (vertical axis) and $\alpha_{\rm c}$ (increasing from left ($\alpha_{\rm c} = 0.0$) to right ($\alpha_{\rm c} = 0.8$)). The parameter $\alpha_{\rm x}$ is set to 0.2 for all the contour plots. The associated total energies were calculated with the DH-RSxc functional (Eq. (7) in main text). For each contour plot the contour line corresponding to 10^{-4} eV is shown with solid colored line. The contour line based on the associated DFT total energies is shown with dashed colored line.

B. Effect of the change of the fraction of the exact exchange in the DH-RSxc-ii functional

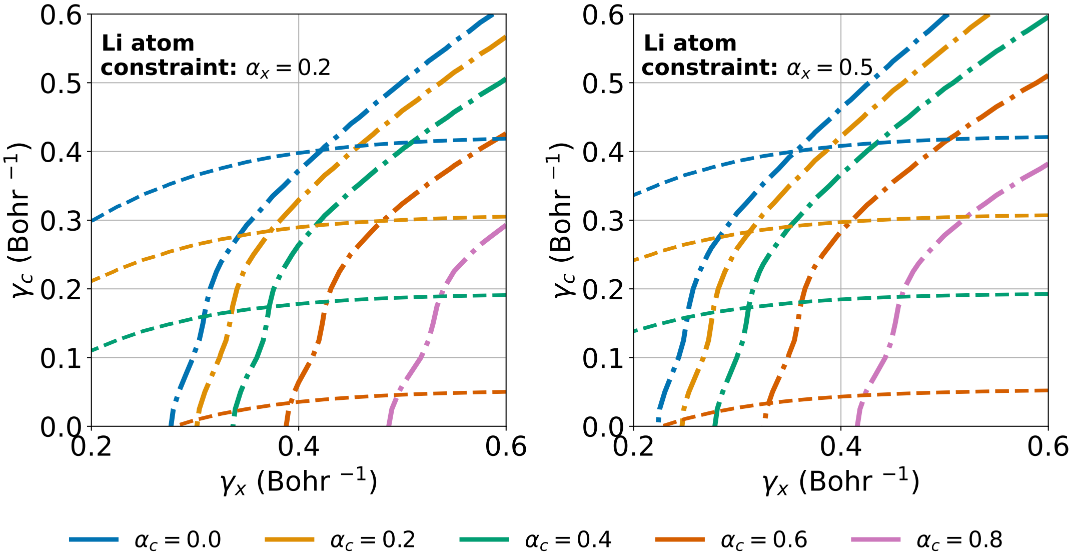

FIG. VI.3. Minimum contour lines for FC (dash-dotted lines) and FS (dashed lines) "middle point" errors equal to 10^{-4} eV for the Li atom. **Left**: DH-RSxc-ii with $\alpha_x = 0.2$, **Right**: DH-RSxc-ii with $\alpha_x = 0.5$

We conclude that the change of the fraction of exact exchange, from 0.2 to 0.5, maintains the overall shape of the middle point curves. It affects the FC curves (as in the single hybrid) more than it does the FS curves. As a direct consequence, the tuned γ_x value is reduced, while the effect on the tuned γ_c value is smaller.

VII. PARAMETERS AND NUMERICAL VALUES FOR FIGS 3, 4

TABLE I. Parameters and errors of the RSH functionals shown in Figs. 3, 4

atom	α_x	γ_x (Bohr^{-1})	α_c	γ_c (Bohr^{-1})	FC (eV)	FS (eV)
			OT-RSH			
H	0.2	1.182	–	–	1.73E−2	3.69E+0
Li	0.2	0.333	–	–	1.08E−2	1.33E+0
B	0.2	0.525	–	–	2.01E−3	2.37E+0
F	0.2	0.642	–	–	−1.78E−3	4.26E+0
			$\gamma_x = \gamma_c$			
H	0.47	1.462	0.2	1.462	−8.67E−5	−7.75E−4
Li	0.62	0.287	0.2	0.287	7.28E−4	−3.50E−3
B	0.62	0.555	0.2	0.555	8.01E−4	−1.92E−3
F	0.53	0.605	0.2	0.605	8.18E−4	8.00E−3
			$\gamma_x \neq \gamma_c$, $\alpha_x = 0.2$			
H	0.2	1.645	0.2	1.460	−4.78E−5	−2.75E−4
Li	0.2	0.372	0.2	0.283	−1.01E−4	8.74E−4
B	0.2	0.761	0.2	0.556	7.34E−4	−2.31E−3
F	0.2	0.893	0.2	0.606	1.97E−3	5.64E−3
			$\gamma_x \neq \gamma_c$, $\alpha_x = 0.5$			
H	0.5	1.434	0.2	1.455	−6.20E−5	6.00E−4
Li	0.5	0.310	0.2	0.280	−4.73E−5	2.40E−3
B	0.5	0.620	0.2	0.546	6.81E−4	5.00E−3
F	0.5	0.631	0.2	0.604	8.79E−4	1.50E−2

VIII. BAR PLOTS AND DISSOCIATION CURVES WITH BLYP-BASED DH DUNCTIONALS

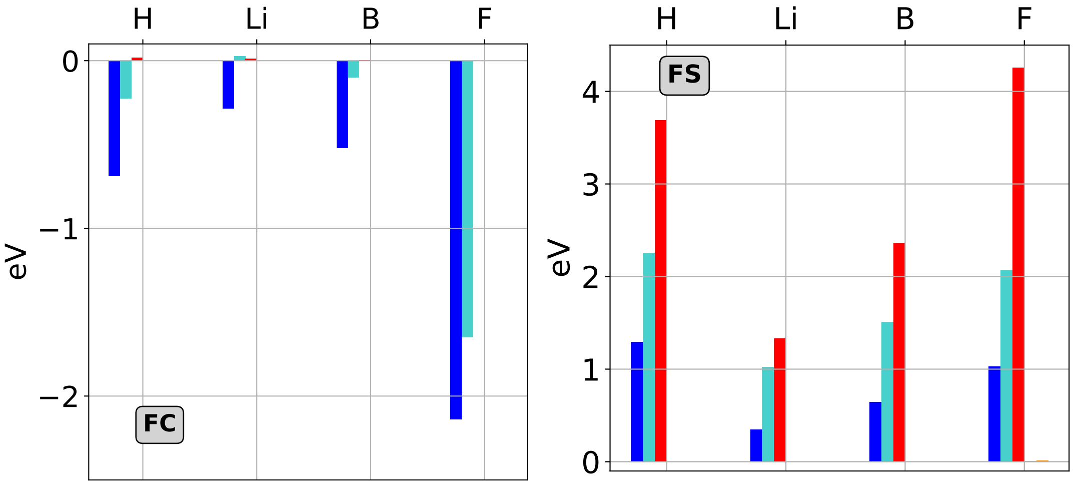

FIG. VIII.1. Middle point **(left)** FC and **(right)** FS error for H, Li, B, and F atoms calculated with B2PLYP (■), ωB2PLYP (■), OT-RSH (■) with $\alpha_x = 0.2$, DH-RSxc (■) with $\gamma_x = \gamma_c$ and $\alpha_c = 0.2$ and DH-RSxc (■) with $\alpha_x = 0.5$ and $\alpha_c = 0.2$. The parameters of the latter two functionals were determined from the intersection points of the middle point FC and FS contour lines, as shown in Fig. 2b-i, and Fig.VI.3 for the Li atom. Some of the functionals exhibit very small errors to see (see Sec. VII for the values of the errors and the parameters of the RSH functionals)

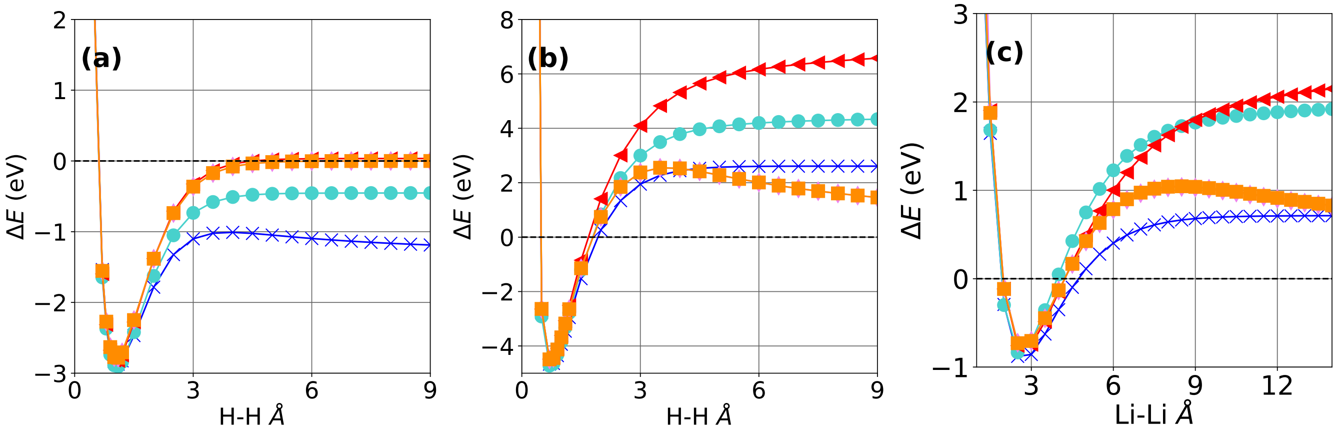

FIG. VIII.2. Dissociation curves of H_2^+, H_2, and Li_2, calculated with the same functionals used in Fig. VIII.1, namely B2PLYP (− × −), ωB2PLYP (−●−), OT-RSH (− ◀ −), DH-RSxc-I (−■−), and DH-RSxc-II (−■−)

Comparison to Figs. 3 and 4 of the main text shows that the corresponding QIDH functionals are generally more accurate.

IX. DISSOCIATION CURVES WITH DH-RSXC-II AND TWO DIFFERENT FRACTIONS OF EXACT EXCHANGE

In Fig. IX.1 we compare dissociation curves obtained with the DH-RSxc-ii functional with $\alpha_x = 0.2$ and $\alpha_x = 0.5$ (see Sec. VII for tuned values). The results are qualitatively identical, which we interpret as a consequence of the tuning of the non-fixed parameters.

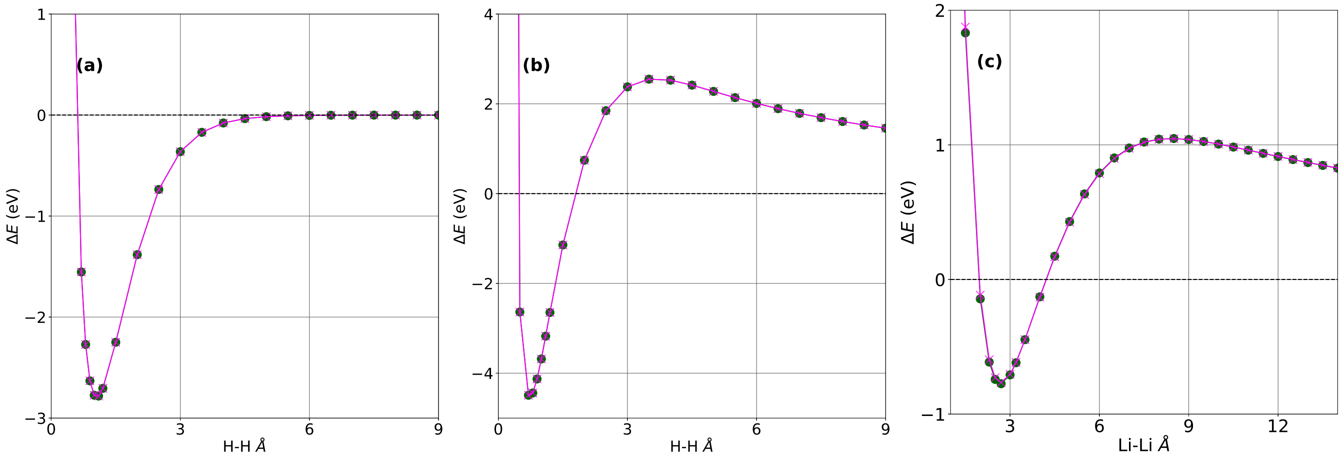

FIG. IX.1. Dissociation curves of H_2^+, H_2, and Li_2, calculated with DH-RSxc-II with $\alpha_c = 0.2$ and $\alpha_x = 0.2$ (—●—) and $\alpha_x = 0.5$ (− × −)

[1] C. Kalai and J. Toulouse, J. Chem. Phys. **148**, 164105 (2018).

[2] Y. G. Khait, J. Song, and M. R. Hoffmann, J. Chem. Phys. **117**, 4133 (2002).

[3] R. R. Li and M. R. Hoffmann, in *Chemical Physics and Quantum Chemistry*, Advances in Quantum Chemistry, Vol. 81, edited by K. Ruud and E. J. Brändas (Academic Press, 2020) pp. 105–141.

[4] S. Hirata and X. He, J. Chem. Phys. **138**, 204112 (2013).

[5] S. Hirata and P. K. Jha, J. Chem. Phys. **153**, 014103 (2020).

[6] J. T. Margraf and R. Bartlett, J. Chem. Phys. **148**, 221103 (2018).

[7] J. Toulouse, F. Colonna, and A. Savin, Phys. Rev. A **70**, 062505 (2004).

[8] J. Toulouse, F. Colonna, and A. Savin, J. Chem. Phys. **122**, 014110 (2005).

[9] S. Paziani, S. Moroni, P. Gori-Giorgi, and G. B. Bachelet, Phys. Rev. B **73**, 155111 (2006).

[10] E. Goll, H.-J. Werner, and H. Stoll, Phys. Chem. Chem. Phys. **7**, 3917 (2005).

[11] H.-J. Werner, P. J. Knowles, G. Knizia, F. R. Manby, and M. Schütz, WIREs Comput Mol Sci **2**, 242 (2012).

[12] H.-J. Werner, P. J. Knowles, G. Knizia, F. R. Manby, M. Schütz, P. Celani, W. Györffy, D. Kats, T. Korona, R. Lindh, A. Mitrushenkov, G. Rauhut, K. R. Shamasundar, T. B. Adler, R. D. Amos, S. J. Bennie, A. Bernhardsson, A. Berning, D. L. Cooper, M. J. O. Deegan, A. J. Dobbyn, F. Eckert, E. Goll, C. Hampel, A. Hesselmann, G. Hetzer, T. Hrenar, G. Jansen, C. Köppl, S. J. R. Lee, Y. Liu, A. W. Lloyd, Q. Ma, R. A. Mata, A. J. May, S. J. McNicholas, W. Meyer, T. F. Miller III, M. E. Mura, A. Nicklass, D. P. O'Neill, P. Palmieri, D. Peng, K. Pflüger, R. Pitzer, M. Reiher, T. Shiozaki, H. Stoll, A. J. Stone, R. Tarroni, T. Thorsteinsson, M. Wang, and M. Welborn, "Molpro, version 2019.2, a package of ab initio programs," (2019), see https://www.molpro.net.

[13] A. D. Becke, Phys. Rev. A **38**, 3098 (1988).

[14] C. Lee, W. Yang, and R. G. Parr, Phys. Rev. B **37**, 785 (1988).

[15] J. P. Perdew, K. Burke, and M. Ernzerhof, Phys. Rev. Lett. **77**, 3865 (1996).

[16] S. Grimme, J. Chem. Phys. **124**, 034108 (2006).

[17] A. Karton, A. Tarnopolsky, J.-F. Lamére, G. C. Schatz, and J. M. L. Martin, J. Phys. Chem. A **112**, 12868 (2008).

[18] A. Tarnopolsky, A. Karton, R. Sertchook, D. Vuzman, and J. M. L. Martin, J. Phys. Chem. A **112**, 3 (2008).

[19] J.-D. Chai and S.-P. Mao, Chem. Phys. Lett. **538**, 121 (2012).

[20] E. Brémond and C. Adamo, J. Chem. Phys. **135**, 024106 (2011).

[21] E. Brémond, J. C. Sancho-García, A. J. Pérez-Jiménez, and C. Adamo, J. Chem. Phys. **141**, 031101 (2014).
